# Supplementary material for: Magnetic Nanoclusters Stabilized with Poly[3,4-Dihydroxybenzhydrazide] as Efficient Therapeutic Agents for Cancer Cells Destruction
Source: Nanomaterials (Basel). 2023 Mar 3;13(5):933. doi: 10.3390/nano13050933 (PMC10005337; doi:10.3390/nano13050933)
Supplement: Supplementary file 1 [file nanomaterials-13-00933-s001.zip › nanomaterials-2255857-supplementary.pdf]

Supplementary Materials

Magnetic nanoclusters stabilized with poly[3,4-dihydroxybenzhydrazide] as efficient therapeutic agents for cancer cells destruction

Ioana Baldea[1], Anca Petran*[2], Adrian Florea[3], Alexandra Sevastre-Berghian[1], Iuliana Nenu[1], Adriana Filip[1], Mihai Cenariu[4], Maria Teodora Radu[2], Cristian Iacovita[5]

1 *Department of Physiology, Iuliu Hatieganu University of Medicine and Pharmacy, Clinicilor 1-3, 400012 Cluj-Napoca, Romania*
2 *National Institute for Research and Development of Isotopic and Molecular Technologies, 67-103 Donat Str., 400293 Cluj-Napoca, Romania*
3 *Department of Cell and Molecular Biology, Faculty of Medicine," Iuliu Hațieganu" University of Medicine and Pharmacy, Pasteur 6, 400349 Cluj-Napoca, Romania*
4 *Department of Biochemistry, University of Agricultural Sciences and Veterinary Medicine, Calea Manastur 3-5, 400658, Cluj-Napoca, Romania*
5 *Department of Pharmaceutical Physics-Biophysics, Faculty of Pharmacy, "Iuliu Hatieganu" University of Medicine and Pharmacy, 6 Pasteur St., 400349 Cluj-Napoca, Romania*
* Correspondence: anca.petran@itim-cj.ro ; Tel: 0040-753-462-246 (A.P.)

1. XPS determination:

Table S1: N1s binding energies and surface chemical states identified from XPS measurements.

	N1s	Chemical state	at. %
	399.4 eV	N-C, NH_2	53.4%
**DHBH@MNC**	400 eV	N-(C=O)-	35%
	401.8 eV	NH^{3+}	11.5%
**PDHBH@MNC**	398.6 eV	N-C, NH_2	41.8%
	399.6 eV	N-(C=O)-	58.2%

2. Heating curves of MNC internalized in cells:

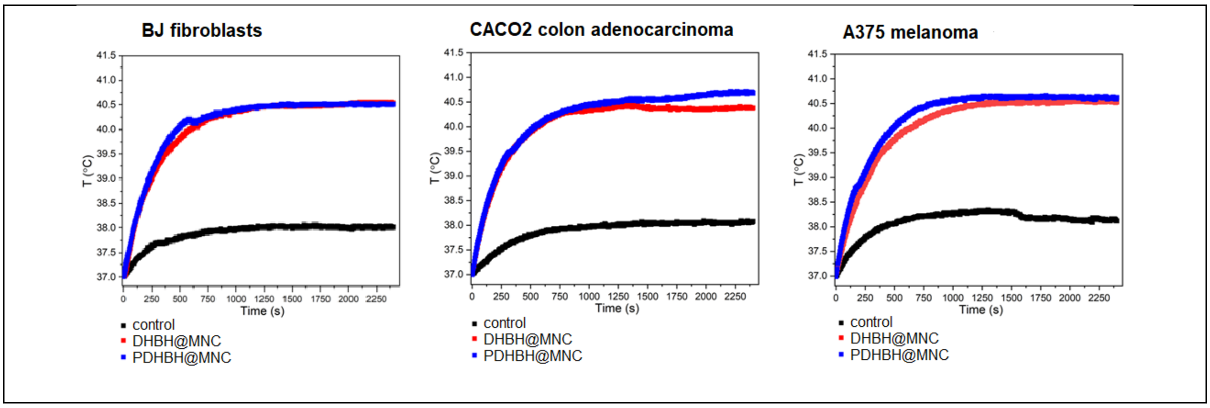

Figure S1. Typical heating curves of cells without and with internalized MNC exposed to an AMF (40 kA/m; 355 kHz) for 40 minutes.

3. Flowcytometric assessment:

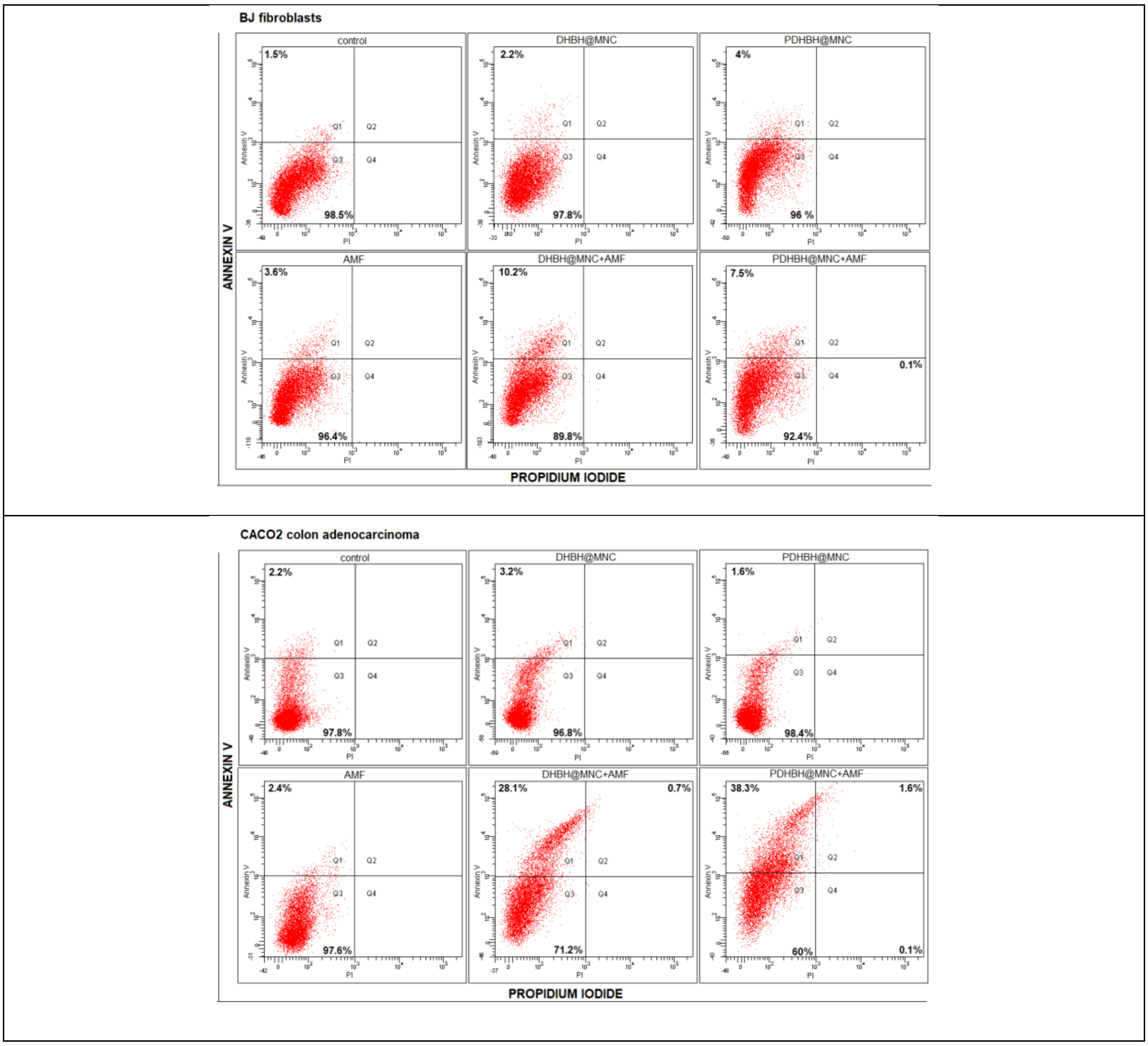

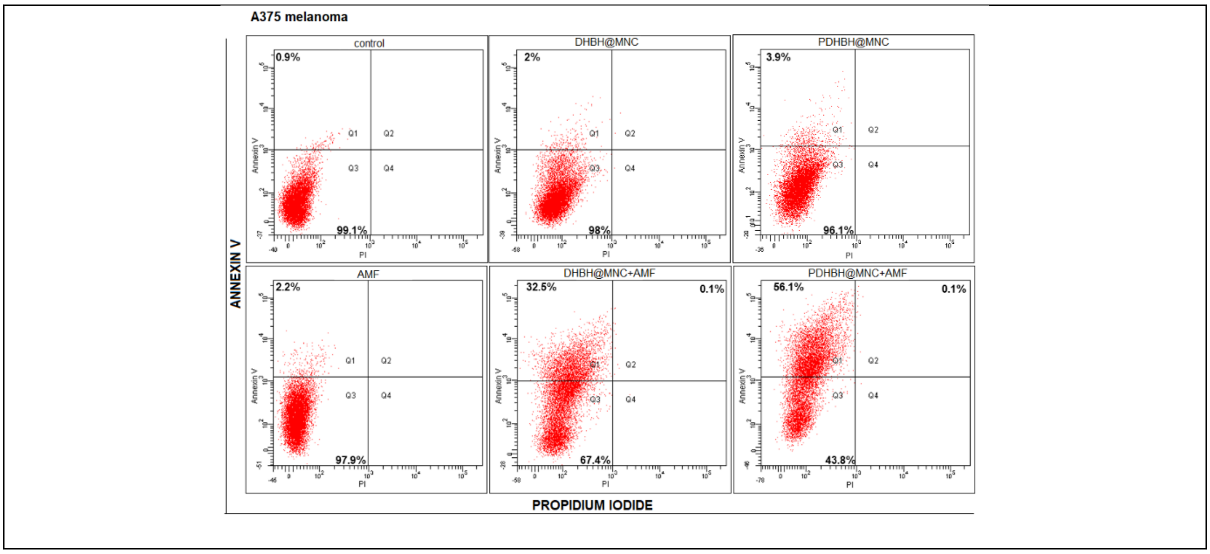

Figure S2. Flowcytometric assessment of the cell death induction by MH exposure: (a) fibroblasts (BJ), (b)-CACO2 and (c) A375 cells . Comparative FACS analysis, quantitative FACS results are expressed as % of total cells counted – viable cells Annexin V (-)/ PI (-) – Q3, early apoptotic cells Annexin V(+) – Q1, necrotic cells PI (+) – Q4, late apoptosis Annexin V (+)/PI (+) – Q2 quadrant, a minimum of 10000 events were quantified for each lot
